# A Case of Double Uterus with a Fœtus in Each

**Published:** 1885-07

**Authors:** W. E. Lane


### A CASE OF DOUBLE UTERUS, WITH A FŒTUS IN EACH.

Under the above caption the *Atlanta Medical and Surgical Journal*, for July, has a paper by Dr. E. W. Lane. It had been read before the Georgia Medical Association, and is a remarkable report of a most extraordinary case, open to sharp criticism, either as to the Doctor's management of the case, or his manner of reporting it, or both.

Dr. Lane relates that on Thursday, Dr. T. J. Hendley was called to a healthy, strong primipara, aged 35, in labor. Dr. H. could not make a diagnosis, but remained with the patient until late Saturday night, making examinations from time to time, till, finally, "he thought he felt the head." He sent for Dr. Lane. Dr. L. arrived Sunday morning, when Dr. H. told him "he thought he felt the toes." Dr. Lane diagnosed a foot presentation, and, with much difficulty, and a fillet, delivered a large male child, dead, from long continued pressure. This was at 1 o'clock, p. m., Sunday. "We were not long in discovering that there was another fœtus in utero," says Dr. Lane. "We ligated below, and left the patient to rest; but as the pains were feeble, and the patient much exhausted, we gave her a stimulant; shortly after which, they began to improve a little. I then made examination by following up the cord. My finger passed into the uterus, but we could find no presenting fœtus; but, being very confident there was another, I again explored the organ, as far as my finger would reach, and, by feeling around, I found a hard substance to my right, and, by further examination, found the os dilated about the size of a twenty-five cent piece, opening laterally into the vagina. We waited all the balance of Sunday, and Sunday night, and until a little after sunrise, Monday morning." [Bear in mind all this time the placenta of the child which had been born Sunday, at 1 p. m. had not been removed.—Ed.] "We waited," the narator goes on to say, "until about 8 o'clock, a. m., (Monday) and finding that the head had become sufficiently low down, and that the fœtus was dead, we punctured its head, [why punctured its head?—Ed.] and I hooked my fingers in the head and delivered a female child of medium size. I introduced my hand, and followed the cord of the last delivered fœtus to the placenta, and delivered it. The uterus contracted as I withdrew it. I then followed the other cord, which lead my hand into another(?) uterine cavity, and found the placenta adherent.'' [i. e. the placenta of the child which had been delivered nineteen hours previously.—Ed.] ''I detached it, and removed it, but the uterus did not contract, and the hemorrhage was considerable.''

Had the womb remained in that condition since the delivery of the fœtus the day before? Was the hemorrhage ''considerable'' all that time? But to resume, and here is the gist of the report, in our opinion:

''I again introduced my hand, and with a cloth in it, about the size of a lady's pocket handkerchief, saturated in apple vinegar, and thoroughly explored the organ; and near the fundus I found a semi-solid substance nearly, or quite, as large as either of the placentæ. I detached and removed it. Uterus No. 2 (?) [this was certainly uterus No. 1.—Ed.] contracted, forcing out my hand with what I took to be a male uterus. Uterus No. 1 (?) was then well contracted, and both could be felt through the walls of the abdomen, distinctly separated, and lying side by side.''

Will somebody tell us what a ''male uterus'' is? or knock us on the head for not knowing? In a general practice of twenty odd years we never came across one, though one would imagine from the light manner with which this one is mentioned, that the woods are full of them, over in Georgia, and the Georgia doctors seem to think no more strangely of finding ''a male uterus,'' a placenta(adherent),and a dead baby in one barrel of a double womb, than would a Texan if told that a rattlesnake, an owl and a prairie dog had been found in the same hole—and all alive! Then, again, the Doctor ''removed'' it—with his hand. The removal of a ''female'' uterus—we must make the distinction now—is a serious matter. We wish, indeed, the Doctor had gone more into a description of this—male uterus—than simply to say it was ''semi-solid,'' and about the size of a placenta or a piece of chalk. Indeed the Doctor mentioned the matter about as he would have done a clot, or a portion of the placenta, or other foreign body. We should have considered a ''male uterus'' worth preserving in alcohol, and sending to the

Smithsonian. We hope our Georgia contemporary will enlighten us further on this subject, for verily our ignorance of such a thing as a "male uterus" is profound.

